# THE ROLE OF PSYCHOSOCIAL AND LIFESTYLE FACTORS IN PROMOTING COGNITIVE HEALTH

**DOI:** 10.1093/geroni/igad104.1302

**Published:** 2023-12-21

**Authors:** Emily Willroth

## Abstract

This symposium will highlight the role of psychosocial and lifestyle factors in cognitive health. Across four talks, we consider a wide range of psychosocial and lifestyle factors including personality, stress, physical activity, and nutrition, and a wide range of cognitive health outcomes including subjective and objective cognitive performance, clinical diagnoses of cognitive status, and cognitive healthspan. First, Dr. Patrick Hill will share findings on links between Big Five personality traits and the degree of concordance between subjective and objective cognitive functioning. Second, Dr. Tomiko Yoneda uses longitudinal data to investigate personality trait predictors of transitions between cognitive states (no cognitive impairment, mild cognitive impairment, dementia) and death, as well as personality effects on cognitive healthspan (i.e., years without cognitive impairment) and total longevity. Third, Nicole Stuart used ecological momentary assessment and activity trackers to investigate links among daily stress, daily physical activity, and daily cognitive performance. Finally, Dr. Julia Sheffler will discuss how we can apply our knowledge of psychosocial and lifestyle factors in cognitive health to inform the development of interventions. She will share quantitative and qualitative findings from proof-of-concept (N=9) and pilot (N=58) studies of a nutrition intervention in older adults at risk for dementia, and she will discuss implications for lifestyle intervention development more broadly. Together, this symposium showcases how a wide range of methods can be employed to reveal the importance of psychosocial and lifestyle factors for promoting healthy cognitive aging.

